# National cervical cancer burden estimation through systematic review and analysis of publicly available data in Pakistan

**DOI:** 10.1186/s12889-023-15531-z

**Published:** 2023-05-05

**Authors:** Novera Chughtai, Kausar Perveen, Sehar Rahim Gillani, Aamir Abbas, Rumi Chunara, Afshan Ali Manji, Salima Karani, Ali Aahil Noorali, Maheen Zakaria, Uzma Shamsi, Uzma Chishti, Adnan A. Khan, Sajid Soofi, Shahid Pervez, Zainab Samad

**Affiliations:** 1 Department of Obstetrics and Gynecology, Aga Khan University, Karachi, Pakistan; 2 Department of Medicine, CITRIC Health Data Science Center, Aga Khan University, 1st Floor Faculty Office Building, Stadium Road, P.O. Box 3500, Karachi, 74800 Pakistan; 3 Department of Medicine, Aga Khan University, Karachi, Pakistan; 4 Department of Biostatistics, School of Global Public Health, New York University, New York, USA; 5 Department of Computer Science and Engineering, Tandon School of Engineering, New York University, New York, USA; 6 Department of Community Health Sciences, Aga Khan University, Karachi, Pakistan; 7 Research and Development Solutions, Islamabad, Pakistan; 8 Department of Pediatrics and Child Health, Aga Khan University, Karachi, Pakistan; 9 Centre of Excellence in Women and Child Health, Aga Khan University, Karachi, Pakistan; 10 Department of Pathology and Laboratory Medicine, Aga Khan University, Karachi, Pakistan

**Keywords:** Cancer, Systematic review, Vaccine, Public health, Screening

## Abstract

**Background:**

Cervical cancer is a major cause of cancer-related deaths among women worldwide. Paucity of data on cervical cancer burden in countries like Pakistan hamper requisite resource allocation.

**Objective:**

To estimate the burden of cervical cancer in Pakistan using available data sources.

**Methods:**

We performed a systematic review to identify relevant data on Pakistan between 1995 to 2022. Study data identified through the systematic review that provided enough information to allow age specific incidence rates and age standardized incidence rates (ASIR) calculations for cervical cancer were merged. Population at risk estimates were derived and adjusted for important variables in the care-seeking pathway. The calculated ASIRs were applied to 2020 population estimates to estimate the number of cervical cancer cases in Pakistan.

**Results:**

A total of 13 studies reported ASIRs for cervical cancer for Pakistan. Among the studies selected, the Karachi Cancer Registry reported the highest disease burden estimates for all reported time periods: 1995–1997 ASIR = 6.81, 1998–2002 ASIR = 7.47, and 2017–2019 ASIR = 6.02 per 100,000 women. Using data from Karachi, Punjab and Pakistan Atomic Energy Cancer Registries from 2015–2019, we derived an unadjusted ASIR for cervical cancer of 4.16 per 100,000 women (95% UI 3.28, 5.28). Varying model assumptions produced adjusted ASIRs ranging from 5.2 to 8.4 per 100,000 women. We derived an adjusted ASIR of 7.60, (95% UI 5.98, 10.01) and estimated 6166 (95% UI 4833, 8305) new cases of cervical cancer per year.

**Conclusion:**

The estimated cervical cancer burden in Pakistan is higher than the WHO target. Estimates are sensitive to health seeking behavior, and appropriate physician diagnostic intervention, factors that are relevant to the case of cervical cancer, a stigmatized disease in a low-lower middle income country setting. These estimates make the case for approaching cervical cancer elimination through a multi-pronged strategy.

**Supplementary Information:**

The online version contains supplementary material available at 10.1186/s12889-023-15531-z.

## Introduction

Cervical cancer is the fourth leading cause of cancer deaths in women worldwide. In 2020 approximately 604,000 women were diagnosed with cervical cancer, and 341,000 died due to the disease [1]. It is estimated that most of these deaths occur in low-and low middle income countries (LI-LMICs) and in the coming years, these countries will bear a majority of the burden of cervical cancer [1]. The identification of a causative association of persistent human papillomavirus (HPV) infection with cervical cancer has led to the development of vaccines to prevent HPV infection and thus cervical cancer. Therefore, HPV vaccination has emerged as a powerful tool to combat cervical cancer. Many high income countries that have implemented population level preventive HPV vaccine and cervical cancer screening programs are well on their way to achieving cervical cancer elimination targets set by the World Health Organization (WHO) but in LI-LMICs, lack of adequate disease burden data hamper important policy-level decisions such as instituting national surveillance to assess progress towards disease elimination and/or implementing cervical cancer prevention and management strategies at the population level [1].

Burden estimates for Pakistan have been sparse and regional and WHO estimates for Pakistan have been estimated using data from neighboring countries and limited data from Pakistan. Because of the absence of comprehensive screening programs at the primary care level, reliable disease burden estimates are lacking. Some estimates that are available to guide policymakers are derived from incident cases presenting at a few select hospitals [1]. Disease burden estimates are important tools to monitor population health, prioritization, health policy, and service planning and therefore a necessary guide for policy makers. We aimed to estimate the incidence of Cervical Cancer in Pakistan.

## Methods

### Systematic review

We conducted a systematic review to assess/ compare the incidence of cervical cancer in different studies and cancer registries (Appendix 1) in Pakistan. The databases used for systematic review were PubMed, Scopus, and Emerald Insight. We searched for published papers pertinent to cervical cancer burden estimation in Pakistan. Inclusion criteria were: 1) English publications in journals between 1995 to 2022, 2) studies reporting on age standardized incidence rate (ASIR) of cervical cancer for Pakistan. In addition to these resources, after talking to the relevant subject matter experts at the national level in Pakistan, different cancer registries and International Agency of Research on Cancer (IARC) websites were accessed for reports and studies presenting estimates on cervical cancer incidence in Pakistan.

**Fig. 1:**
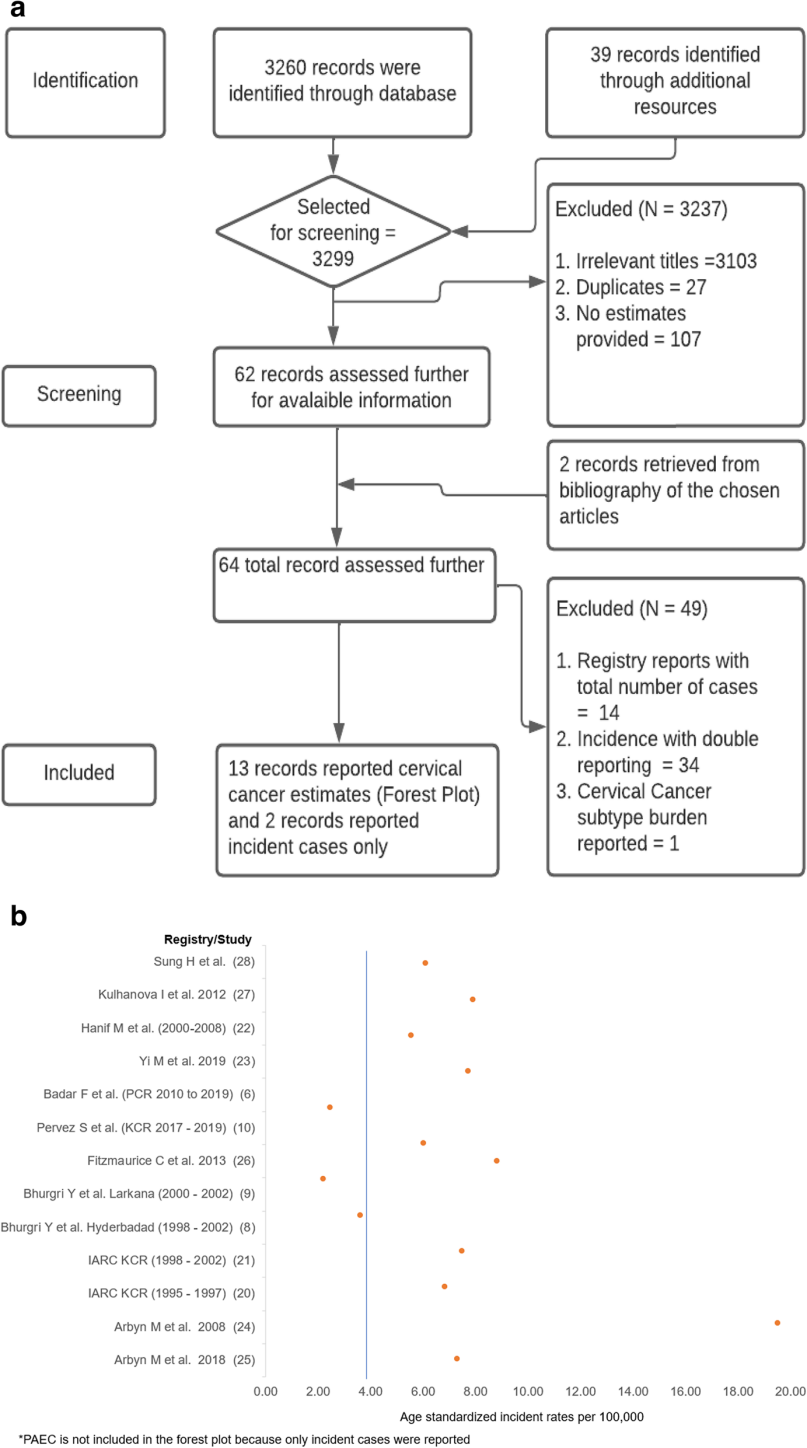
**a** Flow diagram of the systematic review. **b** Age-standardized incidence rates as provided by reports/articles for different cancer registries in Pakistan, and GBD study

**Fig. 2:**
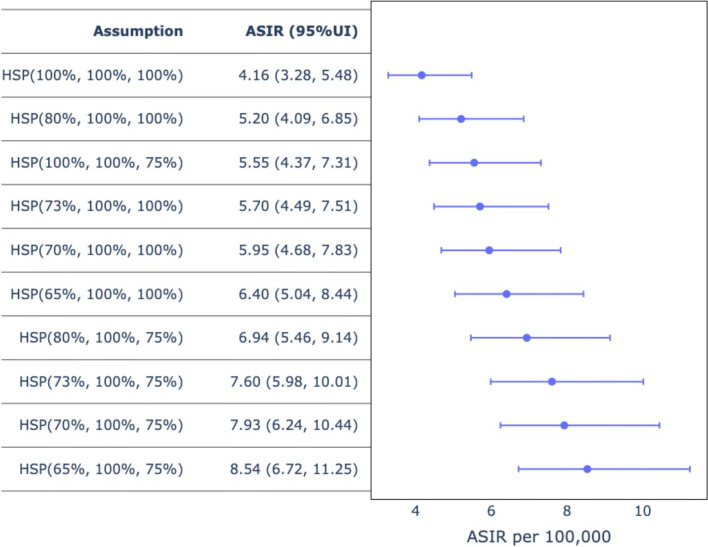
Unadjusted and Adjusted ASIRs [adjustment for varying H (health seeking behavior, S (sensitivity of diagnostic test) and P (probability of appropriate referral or ordering of correct test)]

Search strategy included search terms:

- “Cervical cancer” OR “cervix” OR “cervical neoplasm” OR “cervical dysplasia” AND “epidemiology” OR “cervical incidence” OR “cervical mortality” OR “cervical prevalence” AND “Pakistan”. 1094 articles from identified databases were obtained. Other search terms including: (“cervical cancer”) AND (burden) were also explored separately deriving 2166 articles. Additionally, from the Pakistani cancer registries and IARC websites 39 records were obtained which included reports and study articles published by the different registries. The last date of querying the databases was March 11, 2022. Articles were extracted and reviewed by at least 2 independent reviewers (from SR, AA and ZS) and discordance was resolved through consensus. The 3299 results were further filtered using predefined screening criteria. Irrelevant titles, duplicate studies, studies with no information on cervical cancer, studies with no information on cervical cancer in Pakistan, and studies that provided no estimates of cervical cancer incidence in Pakistan were excluded. This process resulted in 62 specific records (Fig. 1a) and 2 additional records were further retrieved from the bibliography of selected articles, 64 specific records were then further assessed. Registry reports not providing age group wise incident cases, incidence estimates with potential duplication across time periods, and estimates reported on cervical cancer subtype were excluded. The ASIRs from resultant published studies were graphically displayed.

### National burden estimation for Pakistan

Inclusion of data sources for derivation of national estimates:

- We then undertook an analysis of available data in published studies and reports on incident cases in Pakistan between 2015–2019 (Appendix 2). Data were included from higher level registries, while sub-registries that contributed to these higher registries were excluded. For example, Punjab Cancer Registry (PCR) Badar F et al. (PCR, 2010–2012) [2, 3], Badar F et al. (PCR, 2010–2015), [4] and registry reports from Shaukat Khanum Cancer Registry; Mahmood S et al. (SKHRC, 1994–2019), [5] which collaborates with and provides cases to the PCR, were excluded to avoid an overlap of the reported cases being represented in the study.
- PCR published studies for three overlapping time-periods: (PCR, 2010–2012), (PCR, 2010–2015) and (PCR, 2010–2019). Of these, Badar F et al. (PCR, 2010–2019) [6] reported cases for the most extended time-period, and this was selected for inclusion in the merged dataset.
- Similarly, the Dow Cancer Registry; Qureshi MA et al. (2010–2019) [7] has already been collaborating and sharing their data with Karachi Cancer Registry; therefore, these were also excluded. Bhurgri Y et al. (KCR, Hyderabad) [8] and KCR, (Bhurgri Y et al. Larkana) [9] was also excluded as detailed age specific incidence estimates were not provided. We finally selected three registries, Karachi Cancer Registry; Pervez S et al. (KCR, 2017–2019) [10]; Punjab Cancer Registry; Badar F et al. (PCR, 2010–2019) [6]; and Pakistan Atomic Energy Cancer Registry; (PAEC, 2015–2019) [11, 12] that reported cases for the 2015–2019 time period to include in our dataset for estimating the ASIR for Pakistan.

### Statistical analysis

To estimate the incidence using data from these geographically distinct cancer registries (Appendix 3), we pooled cases and applied relevant population denominators adjusted for important variables in the care-seeking pathway [13]. At-risk population estimates were derived from the population within a 15-mile vicinity [14] of the reporting hospitals and adjusted for health-seeking behavior, diagnostic test sensitivity, and the probability of a physician ordering the correct test or making an appropriate referral [15]. Burden estimates from registries may be affected by care-seeking behavior; non-reporting due to misdiagnosis; and physician competence in choosing the correct diagnostic pathway [15]. We modeled our estimates to account for these factors. We adjusted the denominator for health-seeking behavior (*H*), the sensitivity of the test conducted for cervical cancer diagnosis (*S*), and the probability of the appropriate test being ordered (*P*). Since estimates for cancer care-seeking behavior are not available for Pakistan, we modeled three probabilities: 0.80 (approximate health seeking reported for febrile illness), 0.65 (approximate health seeking reported for facility births) and 0.70 (approximate health seeking reported for diarrheal illness) [16]. Given the absence of a cervical cancer screening program in Pakistan, we assumed biopsy as the first diagnostic test, and therefore modeled 100% sensitivity for this test. Based on knowledge assessment studies of cervical cancer, [17] we used 0.75 as the probability of the managing physician ordering the appropriate test. A combination of assumptions were modeled. Because we do not have actual data on the probability of ordering the correct test and health-seeking behavior for cervical cancer in Pakistan and our modeled assumptions for healthcare seeking behavior using other diseases as proxy would still lead to uncertainty in our assumptions, we ran a Monte Carlo simulation giving a probability of 0.75 for ordering the correct test and 0.73 for health-seeking behavior. We used these values to adjust the denominator. The denominator (*D*) was calculated from these parameters and values: Original denominator = D * (H = 1, S = 1, *P* = 1) and New denominator = original denominator * (H = 0.73, S = 1, *P* = 0.75, Uncertainty intervals (95% UI) were also computed for ASIR using the standard error (SE) of the crude incidence rate. Following the WHO GLOBOCAN approach, the standard error was corrected for three major sources of bias: c = data coverage (population weighted average of data coverage area in relation to the estimated area), l = data lag time, and q = data quality. For the sake of simplicity, the three biases have been considered to have the same importance, and a correction based on three categorical variables having the same range of values from 0 to 10 is computed via:

$$SE = se * 100/(100-c) * 100/(100-t) * 100/(100-q)$$

We applied maximum correction for our analysis (c = l = q = 10).

Each reporting registry’s total reported cases were annualized by dividing the number of cases by the reporting period (unless the reported cases were for a one-year duration). The annualized volumes were added to obtain the caseload for the three registries during the 2015–2019 period (KCR did not report cases for 2015–2016). We geo-located the hospitals contributing data to the different registries by mapping their longitude and latitude coordinates and subsequently pinning the locations in OpenStreetMap using ArcGIS version 10.2.2 [18]. We calculated the total 15-mile radius population around these hospitals for five-year age groups (0–4, 5–9, 10- 4… ≥ 70 years). All the 22 hospitals listed under the Punjab Cancer Registry were located in the Lahore district [6]. Due to the potential of overlap between at-risk populations for closely located hospitals in Lahore, we calculated the 15-mile radius population from the coordinate located at the midpoint of all hospitals in the PCR registry. We adopted a similar approach for the Karachi Cancer Registry, which had eight hospitals reporting cases to it [10] (Appendix 3).

First, we calculated age-specific incidence rates per 100,000 for each year within the 2015–2019 period by dividing the summed case volumes by the female population in the 15-mile radius. The incidence rates were then averaged for each age group across the 2015–2019 period to obtain the crude incidence rates. The crude incidence rates were standardized using Segi's world standard population [19] to obtain the age-standardized incidence rates for each age group. The resultant values were then summed to arrive at the age standardized incidence rates for cervical cancer for females in Pakistan.

To estimate the number of new cases of cervical cancer in Pakistan we used the derived unadjusted and adjusted age standardized incidence rates and applied them to the 2020 female population of Pakistan. Outputs were rounded to whole numbers.

## Results

As a result of systematic review we retrieved a total of 15 published articles and reports as shown in Table 1. Out of these 5 local studies i.e. IARC (KCR, 1995–1997), [20] IARC (KCR, 1998–2002), [21] Pervez S et al. (KCR, 2017–2019), [10] Badar F et al. (PCR, 2010–2019) [6] & Hanif M et al. [22] provided incident cases with calculations for ASIR; 2 local studies provided only ASIR estimates but no information on number of incident cases i.e. Bhurgri Y et al. (KCR, Hyderabad) [8] and Bhurgri Y et al. (KCR, Larkana) [9]; 2 local reports i.e. (PAEC, 2015–2017) [11] & (PAEC, 2018–2019) [12] provided information on the number of incident cervical cancer cases alone without estimation of ASIRs. A total of 6 international studies [23–28] provided modeled estimates of cervical cancer incidence for Pakistan. Figure 1b shows the point estimates of age standardized cervical cancer incidence reported by the different records and provided in international studies. Among these Yi M et al. used Global Burden of Disease 2019 data and estimates [29]. Of the studies out of Pakistan based on local registry data, the highest ASIRs were reported by Karachi Cancer Registry across all time periods:1995–1997 ASIR = 6.81, 1998–2002 ASIR = 7.47, and 2017–2019 ASIR = 6.02 per 100,000 women. There was variability in estimated incidence across studies. Modeled estimates for Pakistan published by international studies or consortia reported ASIRs > 6 [23–28].

**Table 1:** Studies identified by systematic review and characteristics addressed in each study; registry, period, author, crude rate and ASIR information used to generate forest plot in Fig. 1

No	Author, Reference no	Registry/ Study Title	Data Source	Time period	Crude rate (per 100,000)	ASIR^a^ (per 100,000)
1	IARC, KCR [20]	Karachi Cancer Registry	Karachi Cancer Registry data from Cancer Incidence Report published on IARC website	1995—1997	3.20	6.81
2	IARC, KCR [21]	Karachi Cancer Registry	Karachi Cancer Registry from Cancer Incidence Report published on IARC website	1998—2002	4.00	7.47
3	Pervez S et al. [10]	Karachi Cancer Registry	Karachi Cancer Registry data	2017—2019	5.14 (estimated)	6.02
4	Bhurgri Y et al. [8]	Karachi Cancer Registry	Hyderabad residents registered at KCR and AKU Pathology collection point in Hyderabad	1998–2002	-	3.60
5	Bhurgri Y et al. [9]	Karachi Cancer Registry	Larkana residents registered at KCR and AKU Pathology collection point in Larkana	2000–2002	-	2.20
6	Hanif M et al. [22]	Institution-based Cancer Incidence in a Local Population in Pakistan: Nine Year Data Analysis	Karachi institute of Radiotherapy and Nuclear Medicine hospital data	2000–2008	-	5.54
7	Badar F et al. [6]	Punjab Cancer Registry	The population-based Punjab Cancer Registry data from PCR website	2010—2019	1.48 (estimated)	2.46
8	PAEC [11]	Pakistan Atomic Energy Cancer Registry	Pakistan Atomic Energy Cancer Registry Report data from PAEC website	2015–2017	-	-
9	PAEC [12]	Pakistan Atomic Energy Cancer Registry	Pakistan Atomic Energy Cancer Registry Report from PAEC website	2018–2019	-	-
10	Yi M et al. [23]	Epidemiological trends of women’s cancers from 1990 to 2019 at the global, regional, and national levels: a population-based study	Global Burden of Disease 2019	1990–2019	-	7.70
11	Arbyn M et al. [24]	Worldwide Burden of Cervical Cancer in 2008	GLOBOCAN 2008 published by IARC	2008	-	19.5
12	Arbyn M et al. [25]	Estimates of incidence and mortality of cervical cancer in 2018: a worldwide analysis	Global Cancer Observatory 2018 published by IARC	2018	-	7.3
13	Fitzmaurice C et al. [26]	The Global Burden of Cancer 2013	Cancer incidence in five continents (CI5) database from IARC	2013	-	8.81
14	Kulhanova I et al. [27]	Profile of cancer in the Eastern Mediterranean region: The need for action	GLOBOCAN 2012	2012	-	7.90
15	Sung H et al. [28]	Global Cancer Statistics 2020: GLOBOCAN Estimates of Incidence and Mortality Worldwide for 36 Cancers in 185 Countries	Global Cancer Observatory (GCO) for 2020	2020	3.00	6.10

^a^*ASIR* Age-standardized rate (per 100,000 population)

Using Pakistan level data combined from 3 registries, we derived an unadjusted ASIR of 4.16 (95% UI, 3.28, 5.48) which translated to an estimated 3376 (95% UI, 2646, 4547) new cases of cervical cancer/year.

**Fig. 3:**
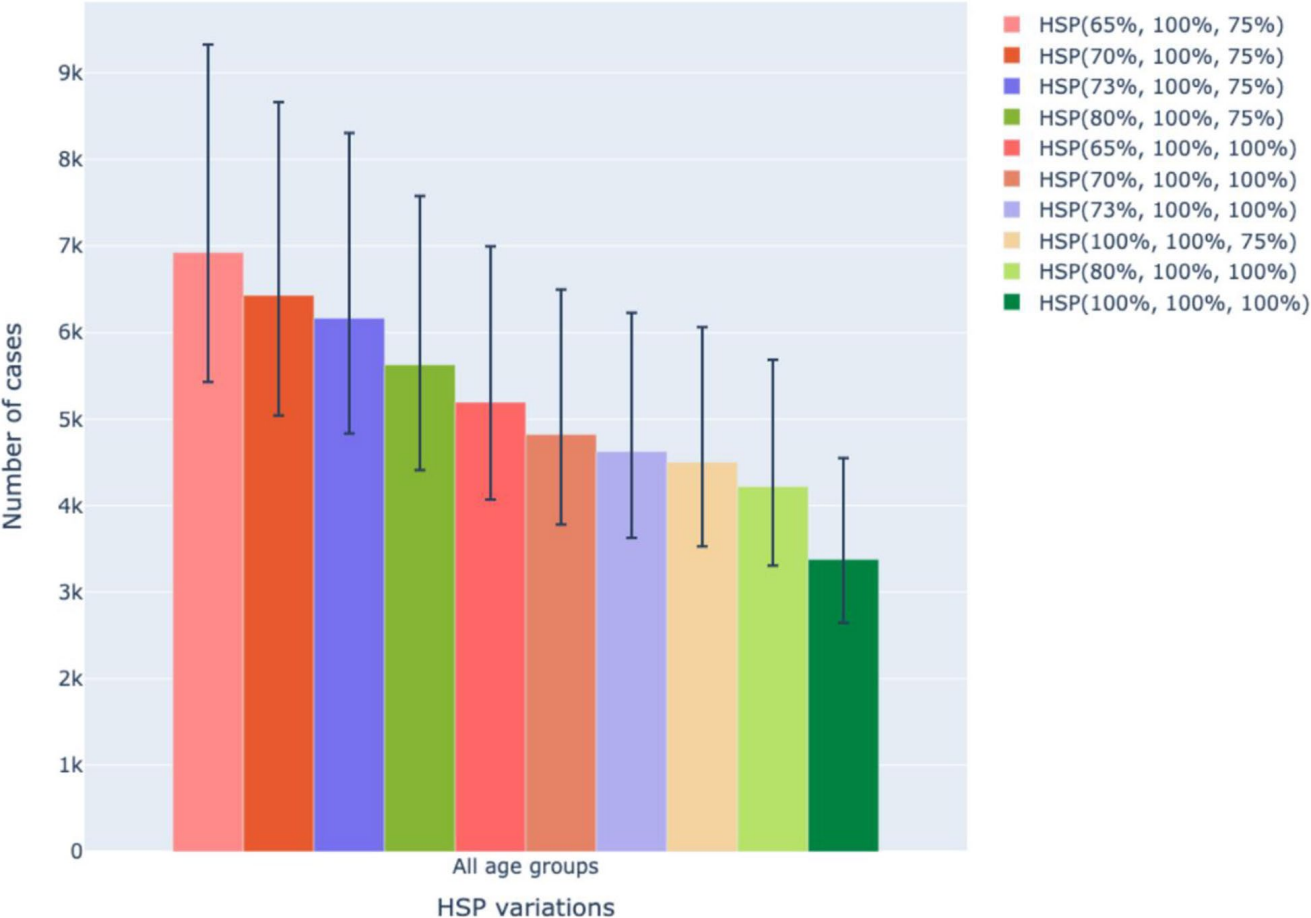
Estimated number of cases with adjusted and unadjusted ASIRs

Using different modeled assumptions, we derived adjusted ASIRs that ranged from 5.20 to 8.54. A maximum adjustment to the denominator yielded an ASIR of 8.54 (95% UI, 6.72, 11.25) translating to an estimated number of new 6925 (95% UI, 5428, 9327) cervical cancer cases per year (Figs. 2 and 3). Using Monte Carlo simulated estimates for model assumptions we derived an adjusted ASIR of 7.60 (95% UI, 5.98, 10.01). The number of new cases calculated based on the adjusted ASIR of 7.60 were 6166 (95% UI, 4833, 8305).

## Discussion

Our main study findings are summarized as follows: 1) We found that there is variability reported in estimates across Pakistan from different geographic locations and for different time periods and that the Karachi Cancer Registry reported the highest incidence rates for cervical cancer in Pakistan over time. 2) Our analysis using combined data yielded unadjusted and adjusted age-standardized incidence rates for Pakistan that are higher than the WHO target for cervical cancer elimination (4 per 100,000 women). 3) We found that incidence estimates were sensitive to adjustments for health-seeking behavior and probability of a physician prescribing the appropriate diagnostic test. These are critical factors that need to be considered especially in the case of cervical cancer, a stigmatized disease in conservative LI-LMIC settings, and should be accounted for in future studies reporting disease estimates. These estimates make the case for approaching cervical cancer elimination through a multi-pronged strategy.

Our literature search revealed that published estimates were based on incident cases presenting at hospitals and the data was then shared with cancer registries. There were no community- or population-level studies, or studies out of a centralized national cancer registry or any regional screening programs in Pakistan [30]. Moreover, we noted that geographically distinct registries reported different ASIRs, with the Karachi Cancer Registry reporting the highest incidence rates (ASIR = 6.02 to 7.47 per 100,000 women) over time. These estimates are in line with estimates derived from the Global Burden of Disease (GBD) data and WHO estimates and well above the WHO 2030 target for cervical cancer elimination set at 4.00 per 100,000 women, hence requiring policy level attention. An analysis of the GBD study data showed an increase in incidence rates in South Asia and a higher incidence in countries with low sociodemographic index (SDI) than those with higher SDI [1]. We noted considerable variability in reported ASIRs for different registries in Pakistan over time. It remains unclear whether true regional variation exists in cervical cancer incidence across Pakistan related to variation in socio-demography. In such a case, a geographically smart strategy may need to be employed towards population-level cervical cancer elimination efforts, with resource allocation for vaccination and screening programs specifically prioritized to areas with an incidence rate above 4/100,000. To comprehend regional variation and disease burden, WHO recommends forming population-based disease registries and acquiring information about cervical cancer mortality from the country’s vital statistics [31].

Our analysis of the combined data from different cancer registries in Pakistan demonstrates the sensitivity of ASIR estimates to the modeled probabilities of healthcare-seeking behavior and probability of physician prescribing the appropriate diagnostic test. Keeping in mind that health-seeking behavior varies substantially based on socioeconomic conditions and the identity of the disease, we modeled the ideal estimates possible for health-seeking behavior. For example, in Pakistan, health care-seeking behavior varies from approximately 70% for diarrheal illness, 66% for deliveries [32, 33] to 80% for fever. Cancer care-seeking patterns are not known and should be sought for future studies, as they are essential for estimating incidence in countries where population level data or large national level registry data do not exist. In conservative settings, cancer care-seeking behavior for female genital tract cancers may be especially low due to poor women empowerment indices where additional barriers to accessing care also exist [34]. Indeed in the Pakistan demographic health survey conducted in 2018, almost 70% of female participants faced at least one of the following problems accessing health care: getting permission for treatment; getting money for treatment; distance to health facility; and not wanting to go alone [17]. We know that healthcare-seeking behavior is an important variable to consider as most cervical cancer patients in Pakistan present at an advanced stage and the associated high mortality is attributable to this late-stage presentation [33, 34]. Though we made assumptions for care-seeking behavior using proxies, the lack of information on health-seeking behavior for cervical cancer and its symptoms remains an important limitation of this analysis. Our estimates for cervical cancer incidence however are plausible because several risk factors appear to be on the rise. These include early sexual debut, a rise in sexually transmitted infections, [30] and smoking. An increase in pre-marital sexual activity and polygamous marriages [35, 36] also contribute to a rise in rates of HPV infection and the associated risk of cervical malignancy [37]. In a study conducted in 2015, Shahid and colleagues noted a high incidence of HPV infection in Pakistani women with pre-cancerous lesions of the cervix [38] and 88% of invasive cervical cancers were found to be associated with HPV 16 or 18. These findings emphasize the urgent need for national-level vaccination and screening programs in Pakistan [39].

Overall, our data points to a substantial burden of cervical cancer in Pakistan. These data signify an urgent need for population-based interventions for disease elimination. The WHO has developed a global strategy to eliminate cervical cancer as a public health problem and has proposed an elimination threshold of 4 cases per 100 000 women. The strategy includes 2030 triple-intervention coverage targets for scale-up of HPV vaccination to 90%, twice-lifetime cervical screening to 70%, and treatment of pre-invasive lesions and invasive cancer to 90% [40]. Several high-income countries have reduced their cervical cancer burden by establishing surveillance and HPV vaccination programs [28, 41]. A recent systematic review and meta-analysis showed a decline in high-risk HPV by 54–83% and precancerous lesions by 31–51% in high-income countries with high vaccination coverage [42]. Consequently, cervical cancer is no longer among the top 20 causes of death in some regions of the world including North America and Australia/New Zealand.

Investments in such interventional approaches will pay dividends in the long run by preventing cervical cancer and reducing its associated mortality. Vaccination coverage remains low (approximately 30%) in LMICs Different approaches have been modeled previously for low-middle income countries. Brisson and colleagues modeled three strategies for cervical cancer elimination in 78 LMICs: girls-only vaccination; girls-only vaccination with one lifetime screening with a Pap smear at the age of 35; and girls-only vaccination coupled with two lifetime screenings at 35 and 45 years. The girls-only vaccination strategy was predicted to reduce incidence rates from 19.8 to 2.1 over the next century, with the potential to avert 61.1 million new cases during this period [43]. A recent comparative modeling study by Portnoy et al. projected the impact of HPV vaccination in Pakistan. The study simulated a vaccination scenario of 90% annual HPV vaccine coverage among 10 cohorts of 9-year-old girls between 2021 and 2030 and estimated that 111,000 to 133,000 cervical cancer cases could be prevented during that time period [44]. Recent work from the Global Burden of Diseases study has suggested cost savings for Pakistan with HPV vaccination with low incremental cost effectiveness ratios noted for the South Asia region [45]. In addition to instituting vaccination programs, it is important to consider the local context. In a conservative society with poor women empowerment indices, a heavily stigmatized disease such as cervical cancer must be carefully addressed through a multipronged approach that includes population-based interventions to increase awareness, behavior change communication, implementation of primary care level screening programs, and national HPV vaccination programs [46, 47]. Designing such an intervention is contingent upon contextual evidence obtained through qualitative inquiries. Therefore, novel approaches such as participatory ethnographic evaluation research may need to be employed. Behavioral change communication can be utilized to increase awareness of cervical cancer, highlight the need for vaccination and aid in improving the uptake of and demand for the HPV vaccine and screening within communities [30]. Pakistan represents substantial ethno-geographic, socioeconomic, and literacy-level heterogeneity therefore, these contextual factors need to be considered when designing interventions. Population-level prevention programs may first need to prioritize regions with a high incidence of cervical cancer along with high HPV vaccine and screening acceptability.

The main limitations to our analysis are inherent to the sparsity of data in general, lack of data on healthcare seeking behavior and on variables in the care seeking pathway for cervical cancer. But the parameters (and their estimates) for adjusting the denominator were obtained through published reports on proxy use cases, Monte Carlo Simulation, and discussions with local subject matter experts. The latter was done to ensure that our assumptions for sensitivity analysis seemed plausible to senior practicing clinicians in this field. The unique aspects and strengths of this work include: geolocating hospitals reporting to registries to calculate denominator populations; combining and analyzing open-source data from geographically distinct cervical cancer registries in Pakistan; and modeling estimates to account for important variables in the care-seeking pathway.

## Conclusion

We found that that the age standardized incidence rates of cervical cancer in Pakistan were higher than the targets recommended by WHO. The cervical cancer ASIR estimates were sensitive to adjustments for health-seeking behavior and the probability of a physician prescribing the appropriate diagnostic test. These factors are particularly pertinent in the context of conservative LMIC settings, where cervical cancer is a stigmatized disease and need to be considered in future studies. Tackling cervical cancer in Pakistan will require targeted screening programs and equitable access to HPV vaccines.

## Supplementary Information

**Additional file 1:** **Appendix 1.** List of cancer registries in Pakistan.

**Additional file 2:** **Appendix 2.** Available data included in combined analysis.

**Additional file 3:** **Appendix 3.** Geospatial map of hospitals covered by PAECR (red), KCR (blue) and PCR (green).

## Abbreviations

ASIR: Age-standardized incidence rate
PCR: Punjab Cancer Registry
KCR: Karachi Cancer Registry
PAEC: Pakistan Atomic Energy Cancer Registry
SKHRC: Shaukat Khanum Cancer Registry
GBD: Global Burden of Diseases
WHO: World Health Organization
IARC: International Agency for Research on Cancer
HPV: Human papillomavirus
LMIC: Low- and middle-income countries
LI-LMIC: Low-and low middle income countries
