# Supplementary material for: National cervical cancer burden estimation through systematic review and analysis of publicly available data in Pakistan
Source: BMC Public Health. 2023 May 5;23:834. doi: 10.1186/s12889-023-15531-z (PMC10163779; doi:10.1186/s12889-023-15531-z)
Supplement: Supplementary file 1 — Additional file 1: Appendix 1. List of cancer registries in Pakistan. [file 12889_2023_15531_MOESM1_ESM.pdf]

Supplemental Material

Appendix 1. List of Cancer Registries in Pakistan

1. Karachi Cancer Registry
2. Punjab Cancer Registry
3. Pakistan Atomic Energy Cancer Registry
4. Shaukat Khanum Cancer Registry
5. Dow Cancer Registry
6. Aga Khan University Hospital Cancer Registry
