# Supplementary material for: National cervical cancer burden estimation through systematic review and analysis of publicly available data in Pakistan
Source: BMC Public Health. 2023 May 5;23:834. doi: 10.1186/s12889-023-15531-z (PMC10163779; doi:10.1186/s12889-023-15531-z)
Supplement: Supplementary file 2 — Additional file 2: Appendix 2. Available data included in combined analysis. [file 12889_2023_15531_MOESM2_ESM.pdf]

Appendix 2. Available Data included in Combined analysis

No.	Registry	Data Source	Population	Denominator	Time Period	Age Category	Links
1.	Pervez S et al.(10)	Study Article	8 private and public sector institutes from all over Karachi	Karachi Female Population	2017-2019	0-14, 15-19, >20 years	https://pubmed.ncbi.nlm.nih.gov/33247682/
2.	Badar F et al. (6)	Study Article	22 Collaborating Centers from the district of Lahore	Lahore District	2010-2019	5-year age groups; 0-75+	https://bmjopen.bmj.com/content/11/8/e047049
3.	(PAEC)(11)	Registry Report	18 hospitals from all over Pakistan	-	2015-2017	5-year age groups; 0-85+	https://paec.gov.pk/Documents/Medical/PAECR_report_2015-17.pdf
4.	(PAEC)(12)	Registry Report	18 hospitals from all over Pakistan	-	2018-2019	5-year age groups; 0-85+	https://paec.gov.pk/Documents/Medical/PAECR_report_2018-19.pdf
