# Supplementary material for: National cervical cancer burden estimation through systematic review and analysis of publicly available data in Pakistan
Source: BMC Public Health. 2023 May 5;23:834. doi: 10.1186/s12889-023-15531-z (PMC10163779; doi:10.1186/s12889-023-15531-z)

Appendix 3. Geospatial map of hospitals covered by PAECR (red), KCR (blue) and PCR (green).

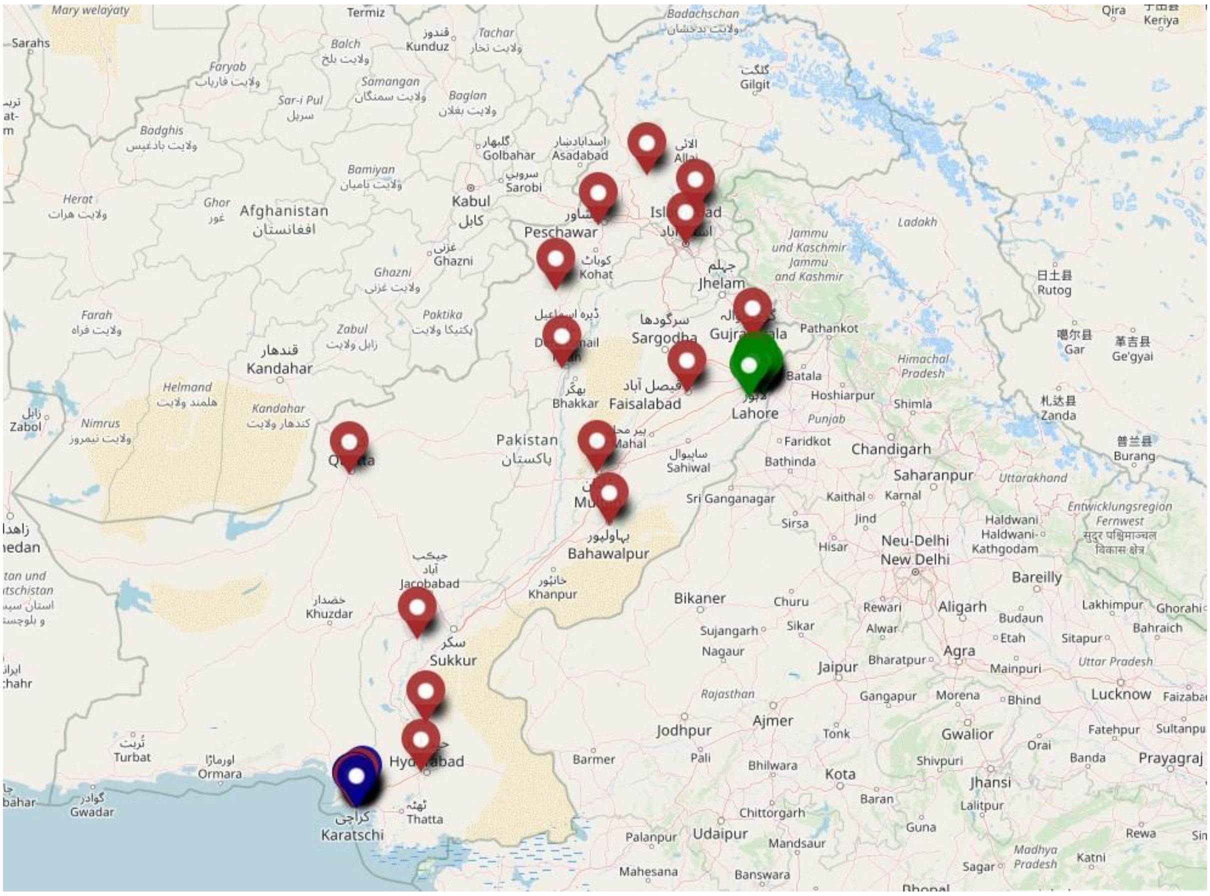

Supplement: Supplementary file 3 — Additional file 3: Appendix 3. Geospatial map of hospitals covered by PAECR (red), KCR (blue) and PCR (green). [file 12889_2023_15531_MOESM3_ESM.pdf]
